# Short-Term Behavioural Responses of Impalas in Simulated Antipredator and Social Contexts

**DOI:** 10.1371/journal.pone.0084970

**Published:** 2013-12-20

**Authors:** François-René Favreau, Olivier Pays, Anne W. Goldizen, Hervé Fritz

**Affiliations:** 1 School of Biological Sciences, University of Queensland, Brisbane, Australia; 2 Laboratoire de Biométrie et Biologie Evolutive, UMR-CNRS 5558, Université Claude Bernard Lyon 1, Villeurbanne, France; 3 Groupe Ecologie et Conservation, Université d’Angers, Angers, France; 4 CNRS HERD Project, Hwange National Park, Zimbabwe; University of Tasmania, Australia

## Abstract

Prey animals often have to trade off foraging against vigilance. However, vigilance is costly and individuals are expected to adjust their vigilance and its cost in relation to social cues and their predation risk. To test this, we conducted playback experiments in the field to study how lions’ (*Panthera leo*) roars and male impalas’ (*Aepyceros melampus*) territorial vocalizations affected the vigilance and foraging behaviours as well as movements of female impalas. Our results show that impalas adjusted their activities in different ways depending on the vocalizations broadcast. After lions’ roars were played, female impalas increased their vigilance activity (in particular increasing their high-cost vigilance – vigilance without chewing), decreased their bite rates and increased their movements, whereas male impalas’ vocalizations caused females to decrease their vigilance (decreasing their low-cost vigilance – vigilance while chewing) and increase their movements without affecting their bite rates. Therefore, it appears that predators’ vocalizations stimulate anti-predator behaviours such as vigilance and movement at the expense of foraging, whereas males’ vocalizations increase individuals’ displacements at the expense of vigilance. Overall, this study shows that both predator and social cues have direct effects on the behaviour of gregarious prey and need to be considered in future studies.

## Introduction

Predators have profound effects on prey species, affecting their abundance, distribution (e.g., [1,2]) and many aspects of their behaviour, such as the time spent in vigilance and foraging or movements either within or between foraging areas [3–5]. Prey animals living in groups also adjust their vigilance to the presence of other conspecifics [6,7]. Such social monitoring allows prey to assess predation risk from alerted companions [8], monitor competitors [9], gain information about food patches [10] or maintain group cohesion [11]. However, the relative proportions of vigilance time devoted to antipredator versus social vigilance vary among species, probably due to differences in species’ vulnerabilities to predation and differences in their social behaviours. In addition, to satisfy their metabolic requirements, prey must also spend most of their active time searching for food [12], creating a trade-off between foraging and vigilance [13]. In the literature, the study of this trade-off has often only considered vigilance as an antipredatory behaviour, thus underestimating the part of this activity that is dedicated to monitoring conspecifics (e.g., [14,15]). It is thus important to understand how social foragers, such as many herbivores, adjust their trade-off between foraging and the use of vigilance for antipredator and social purposes. 

The cost of vigilance can be reduced when animals are able to be vigilant while handling their food; for example, herbivores can devote a part of their chewing time between bites to vigilance activity and thus limit the reduction of their food intake [16–18]. The ability to be vigilant while chewing could be particularly advantageous to herbivores, as their short-term food intake is often limited by chewing and swallowing rates rather than by the rate of encountering food [19]. Recent studies have thus distinguished between vigilant bouts when the animal is standing alert without chewing its food (hereafter called “exclusive vigilance”) and vigilant bouts when the animal is vigilant while chewing its food (hereafter called “vigilance while chewing”) (e.g., [16,17] ). Fortin et al. [16] showed that an increase in the biomass of food decreased herbivores’ bite rates because animals took larger bites, which took longer to chew, allowing them to increase their time in vigilance while chewing. However, the immediate benefit of such vigilance/feeding multitasking could be reduced if the quality of predator detection is lower as a result of vigilance while chewing compared to exclusive vigilance [20]. If this was the case, prey would be predicted to use relatively more exclusive vigilance than vigilance while chewing for antipredator versus social vigilance; to date it remains unknown whether this is the case. 

The vigilance responses of prey in relation to the risk of predation have often been investigated in relation to predator densities or by using proxies of perception of predation risk (e.g., [17,21]); such studies have usually shown that foragers increased their vigilance activity when their perceptions of predation risk likely increased. The presence of predators has also been simulated experimentally by exposing prey to predators’ vocalizations; even though most predators are silent while hunting, playbacks of their vocalizations have appeared to suggest the predator’s presence in the surroundings to the prey and led to an increase in the vigilance activity of the prey (reviewed by [22]). Contrasting results have been reported on the effects of predator playbacks on movements of prey within a foraging patch. Caro et al. [23] reviewed the antipredator behaviour of 200 prey species and reported that while some species tend to freeze or remain motionless when a potential predator is detected, many others increase their movements to escape, bunch, inspect, or even to attack predators. Multiple methods have also been used to examine how prey animals adjust their vigilance to the presence of conspecifics. Studies have investigated the relationships between vigilance and distance to the nearest neighbor [24], and the number [25] and behaviours of other group members [26]. Moreover, as many species communicate using vocalizations, playbacks of social calls can be useful for investigating the effects of social events on vigilance and foraging activities. However, the effects of non-alarm social calls on the vigilance of conspecifics have only been demonstrated in a few species, including marmosets (*Callithrix kuhlii*) [27], meerkats (*Suricata suricatta*) [28] and topi antelopes (*Damaliscus lunatus*) [29]. In some ungulate species such as impala (*Aepyceros melampus*) and red deer (*Cervus elaphus*), males can be highly vocal when defending their territory or their harems during and prior to the breeding period (e.g., [30,31]). In this context, McComb (1991) observed that the roars of male red deer, which were displayed during the breeding period when they gathered and defended harems, induced vigilance and attracted females. While conspecifics can clearly disturb foraging activity (e.g., through competition), the foraging costs associated with social vigilance remain largely unknown. The use of comparable playbacks of the calls of predators and of conspecifics provides a strong design for comparing the relative uses of exclusive vigilance and vigilance while chewing in antipredator versus social contexts. 

We used playback experiments to investigate how lions’ roars and impalas’ social vocalizations affect different aspects of female impalas’ behaviour. We quantified vigilance (including the separate use of exclusive vigilance and vigilance while chewing), bite rates and step rates of females before and after carrying out playbacks of lions’ roars, male impalas’ territorial vocalizations and the sounds of common birds of the area as a control. We chose to use playbacks of non-threatening common bids of the area as controls instead white noise at the same intensity as that of the lion and impala playbacks because we wanted the control trials test for responses to the presence of the observers and playback equipment, but without causing significant additional perturbations for the animals. To mimic the natural levels of the particular sounds used, the control playbacks were played at lower intensities compared to the ones of lions and male impalas. If impalas reacted to all three playback types indiscriminately, their post-playback behaviour would be indistinguishable. If they reacted to the volume only, their responses would be greater to the playbacks of lions and impalas but without any difference between those. Finally, if they reacted to the particular calls, they would be expected to respond weakly or not at all to the control playbacks, and to respond to both the lions’ and impalas’ playbacks but in different ways. 

We predicted that female impalas would react to the particular playbacks played and would increase their time spent in vigilance after both the lion and impala playbacks but with a much stronger response after lions’ roars. Indeed, we predicted that they would show an increase in vigilance after lions’ roars mainly due to exclusive vigilance as individuals are expected to focus on their survival under such an immediate threat and thus to use the most effective form of vigilance. In addition, after males’ vocalizations, female should also increase their vigilance to gather information about the behaviours of conspecifics. However, we predicted that this increase in vigilance would be mainly due to an increase in vigilance while chewing as those vocalizations are not associated with actual danger. We also predicted that after hearing both types of vocalizations, but especially lions’ vocalizations, individuals would reduce their bite rates as a consequence of their higher investment in vigilance. Finally, we expected females to increase their movements (measured as step rates) after being exposed to both lion and impala vocalizations as these reactions have been previously reported in other ungulate species.

## Methods

### Study Site, Study Species and Population

The experiment was conducted around the Main Camp area in Hwange National Park (HNP) (19°00’S, 26°30’E) on the north-west border of Zimbabwe. The study site is composed of an open grassland area of 64 ha surrounded by bushes (*Acacia*/*Combretum*). Data were collected from the beginning of March to the end of April 2012 during the end of the wet season, which occurs from the end of October to the end of April, and prior to the rutting period, which lasts from the beginning of May until mid-June [32,33]. During this period, impala females occurred in fairly large but regularly changing herds that moved through the territories of dominant males, which consequently spent much energy defending their territories and trying to keep herds within their boundaries [34,35]. Territoriality was observed prior to the rutting period from February [36]. Territorial males displayed many territorial behaviors; they advertised their presence using static postures, defecating and urinating in dung patches, depositing smelly secretions to mark the area, and using loud territorial vocalizations [37]. Territorial vocalizations are composed of snorts and roars. They are used in various situations, such as indicating to potential rivals the holding of a territory, but also during agonistic interactions against other males or during the chasing of subadult males out of the herd and the chasing of females trying to escape from the male’s territory [30,32–34].Female impalas from this area spent on average 14% of their time in vigilance in a previous study, with 81% of this in vigilance while chewing; this time allocation was resource-dependent (i.e. varied with grass height) [17]. Impalas are characterized as mixed-feeders, as they alternate between grazing in the wet season and browsing in the dry season because of changes in food quality [38]. During this study (end of the wet season) impalas were mostly grazers. Pays et al. [17] reported that the grass biomass available to the impalas on the study area during this season in 2009 varied between 20 and 150 g/m^2^. An increase in food biomass within this range of values leads to an increase in intake in selective herbivores of similar body sizes (sheep, *Ovis aries*, [19]; Thomson’s gazelles, *Gazella thomsoni* [39]). Impalas’ bite rates have only been described for limited measures of biomass (or sward height) by Okello et al. [40] but are comparable to those exhibited by Thomson’s gazelles [39]. 

We observed 50 to 100 females daily at the study site, mainly foraging (mostly grazing) in the open field. All female impalas in the study area formed a single clan that was divided into a variable number of herds with marked fusion–fission dynamics; this was known because about 30 adult females were ear-tagged. Although our study was conducted during the two months immediately prior to the rutting period, we observed the territorial male every day, usually with the biggest group of females. The female herds were composed of a majority of females but also included juveniles. We did not observe young males in the herds and supposed that they had already been evicted by the territorial male. The dominant male was occasionally observed or heard displaying territorial behaviours and chasing escaping females. In the Main Camp area, impalas have multiple predators, including lions, spotted hyenas (*Crocuta crocuta*), leopards (*Panthera pardus*), cheetahs (*Acinonyx jubatus*) and African wild dogs (*Lycaon pictus*). 

### Ethics Statement

Our experiments complied with the current laws of Zimbabwe. They were conducted under permits from the Director General of the Zimbabwe Parks and Wildlife Management Authority and approved by the Native and Exotic Wildlife and Marine Animals Ethics Committee of the University of Queensland (AEC Approval Number: SBS/358/11/HERD). While we wanted a suitable number of observations to achieve robust results, we limited the number of playbacks as much as possible to minimize their impacts on the impala population and on other mammals that were in the area. In savanna ecosystems, it is known that such playbacks can attract resident lions [41], although we never experienced this situation during our experiment.

### Playback Stimuli

We observed the effects of playbacks of lions’ and of male impalas’ vocalizations on foraging females. Even though lions are quiet while hunting, we felt that playbacks of lions’ roars would simulate their presence in the vicinity, as lions of both sexes usually roar to signal territory occupancy or to contact pride members [42]. To test the reactions of impalas to social stimuli, we exposed females to male impalas’ territorial vocalizations, which were composed of snorts and roars as described above. In addition, to control for possible perturbations from the presence of the equipment or the experimental design, we played familiar and non threatening songs of birds as control stimuli. These types of controls are often used in playback experiments (e.g., [43,44]). We used songs of the red-eyed dove (*Streptopelia semitorquata*) and the piping Cisticola (*Cisticola fulvicapilla*). We checked that there were no differences between the pre- and post-playback phases in the behaviour of impalas to these control playbacks, to determine the robustness of our neutral context control. 

To avoid problems of pseudoreplication, we used three different exemplars of each stimulus that were chosen randomly for each trial. Recordings used included our own local recordings and recordings from different commercial sound archives from South Africa and Zimbabwe. Both lions’ roars and male impalas’ roars can vary in duration; lions’ roars have been measured to last from 17 to 90 s according to Stander and Stander [45]. At our field site, roars by male impalas in the presence of females (mostly chasing females or subadult males) never exceeded one minute and mostly lasted around 20 s (H.F. and O.P. Pers. Obs.). We chose to standardize our playbacks to a plausible duration, but to make them not too long to minimize disturbance and habituation. Therefore, to have comparable stimuli, we selected and edited 15 seconds of each recording for each type of playback using the software program Audacity 1.2.6. All the playbacks were then transferred to an iPod Classic (Apple Inc., Cupertino, California) in the AAC format and played through a powered portable speaker (Megavox pro 6000, Anchor, Carlsbad, California). All playbacks were calibrated by ear at levels perceived by the experimenters as ‘natural’ for each source species in order to be perceived as realistic by the animals; this method has been used in several playback experiments done in the field (e.g., [46,47]). The sound levels of the playbacks of the lions’ roars and impalas’ territorial vocalizations were quite similar, while the control playbacks had lower intensities.

### Experimental Protocol

To minimize possible habituation of the animals, we used a random rotation every three days, including two days with playbacks and one day without. We also limited the number of playbacks to two per day: one control stimulus and either one lion’s roar or one impala’s call. The control playback sequence was always played first, as we assumed that the control playbacks would not (or would only weakly) affect the animals’ behaviour. We left about 30 minutes between the end of the control trial and the beginning of the next one. In addition, for each day of playbacks, the type of sound (lion or impala) played after the control and the versions of the sounds were chosen randomly. A previous study reported that different species of ungulates, including impalas, returned to their baseline behaviour within a few seconds to a minute after being exposed to baboons’ alarm and contest calls [48]. Therefore, thirty minutes would have been more than sufficient time for the animals to return to normal activity if they had been disturbed by the playbacks of bird songs. To make the behavioural context as realistic as possible, all the observations were made in the late afternoon (17:00 to 19:00), when it is common to hear lions roaring and males displaying territorial vocalizations. However, playbacks were only broadcast if we had not heard either a lion roaring or a male vocalizing on the study site during the 60 minutes preceding the trials. We used one car hidden behind trees or bushes and parked approximately 100 meters from the focal group to play the sounds, and a second car that was not hidden to film the animals from a distance of 50 to 100 meters. The impalas were habituated to cars in this area as studies had been carried out there for many years, and our presence did not seem to affect their behaviour. In order to improve our sample size and reduce the animals’ habituation, two or three observers filmed different focal animals during each trial (video cameras: Sony DCR-SR30, 20× optical zoom; and Sony DCR-HC51E, 40× optical zoom, Sony Corporation, Tokyo, Japan); we dealt with the possible pseudoreplication this may have caused by including the group identity in the analysis. We waited for all group members to be relaxed (defined as the females feeding, grooming or looking around while chewing, and the males not chasing females or making noise), each observer focused on a focal foraging female and then we started the trial. Several individuals were filmed during the same session on some days and not all sampled individuals were marked; however, we took care to avoid filming the same individual twice during the same afternoon. We cannot be sure that we did not film the same individuals on different days. However, as there were over 100 different individuals using the study area we believe that there will have been little pseudo-replication and this would not have affected our results. 

We started each trial by filming the animals for 3 minutes before playing the sound (pre-playback period) and continued filming during the 15 seconds playbacks and for another 3 minutes immediately after the playbacks (3minutes post-playback period). For the analyses, we retained only the behavioural sequences of females in groups whose sizes and compositions did not vary during the trials. We defined group size using a maximal distance of 50 meters between adjacent individuals and on the basis of the maintenance of social and spatial cohesion of group members during the observation (as in [17] on the same species). The different observers took care not to film the same animal and to switch to another focal animal for the second trial when two sounds were played on the same day. To do so, the observers filmed the impalas in one part of the group during the first trial and then in a different part during the second trial. The observers waited at least 30 minutes before starting a second trial; during these 30 minutes they were watching the group to see if it was moving. On the rare occasions when the group had moved, the observers changed to another group of individuals to perform the second trial.

For each observation, the observers recorded the date, group size and distance to cover (i.e. distance between the focal female and the closest bushes that would have hidden animals of the size of the impalas or their predators: 0-25, 26-50, 51-100, 101-200, more than 200 m). The observers also recorded grass height using three categories: *short grass* (below impala’s hooves), *medium grass* (below the upper part of the metacarpals), and *tall grass* (when grass height reached the tibia). Trials were not conducted when wind speeds were high, and we positioned the speaker up wind in order to send a clear signal to the animals. 

An animal was considered vigilant when it raised its head above the horizontal, looking around without moving its feet. We also distinguished between two types of vigilance. We considered an animal to exhibit vigilance while chewing when it raised its head while chewing and exclusive vigilance when it raised its head without chewing. If an animal engaged in both types of vigilance during the same bout, we measured the times allocated to each activity separately. Bites were easily observable and counted through repetitive movements of the head, and steps were counted as forward movements of the left front leg.

We sampled the responses of 31 females to playbacks of lions’ roars, 35 females to playbacks of male impalas’ calls and 45 females to the control stimuli during 15, 15 and 31 playbacks, respectively. These playbacks were done during 29 observation sessions.

### Data Analyses

To study the responses of the focal female impala to the different stimuli, we extracted from the video sequences the proportions of time spent by females in all vigilance, and in exclusive vigilance and vigilance while chewing separately, during the pre- and the post-playback periods. We also quantified the numbers of bites taken per minute (bite rate), and the numbers of steps per minute (step rate) performed by the focal animals during both periods. 

To test the effects of the different stimuli on the vigilance, foraging and movements of female impalas, we used linear mixed-effects models for paired samples with (1) the total proportion of time spent in vigilance, (2) the bite rate, and (3) the step rate as the dependent variables and the time periods (pre-playback and post-playback), the types of playback played (Control, Lion and Impala) and their two-way interactions, as independent variables, and individual identity (to pair the samples) within group identity as two nested random factors. The group identity variable controlled for the pseudoreplication caused by studying multiple focal females in the same group at the same time. We also included date to control for possible habituation, group size, distance to cover and grass height in the models as control factors as they might have influenced impalas’ behaviour. To achieve homoscedasticity and normality, the proportions of time spent in vigilance were arcsine-square-root transformed and the numbers of steps per minute were log-transformed.

We further investigated the effect of the different stimuli on exclusive vigilance and vigilance while chewing separately in order to determine whether the females’ short-term responses to the playbacks involved different levels of use of these two types of vigilance. Due to statistical constraints (in particular to fulfill normality of the response variables), we could not examine the proportion of time an impala spent in each type of vigilance (as it was used for the total time spent in vigilance). To test the effects of the stimuli on the vigilance while chewing, we used the log-transformed time that a female impala spent in vigilance while chewing allowing us to use linear mixed-effects models with the procedure as described above. As many females did not exhibit exclusive vigilance, we had many zeros in the data set (for both pre- and post-playback periods) and the log-transformation failed to work. We thus used zero-inflated Poisson mixed-effect models (on non-transformed data) that dealt with the zero-inflated Poisson distribution of the time spent in exclusive vigilance. Here we compared a mixed-effects model including the total time spent in exclusive vigilance as the dependent variable and the time periods and the types of playback played as independent variables, including group identity as random factors and the same model including the interaction between the time period and the type of playback played using a log-likelihood ratio test. When an interaction between two variables significantly affected the response variable, we performed a post-hoc test including the Holm correction to counteract the problem of multiple comparisons. 

The statistical analyses were performed with R 2.13.1 (R Development Core Team 2011).

## Results

### General Behavioural Responses to the Playbacks

Overall, we found significant effects of the interactions between the playbacks and the time periods on the total vigilance, vigilance while chewing, exclusive vigilance, bite rates and step rates of female impalas, indicating different behavioural responses before and after the stimuli in relation to the playbacks played (Table 1).

**Table 1:** Effects of time period, type of playback and their interaction on the proportion of time spent in vigilance, the bite rate, the step rate, the time spent in vigilance while chewing and the time spent in exclusive vigilance, controlling for the effects of date, group size, distance to cover, and grass height.

**Activity**	**Variables**	**numDF**	**denDF**	**F-value**	**p-value**	**Coeff ± SE**
Vigilance	(Intercept)	1	100	614.309	< 0.001	0.288 ± 0.022
	Time period	1	100	0.028	0.867	See Table 2
	Playback	2	42	3.628	0.035	See Table 2
	Time period × Playback	2	100	4.631	0.012	See Table 2
Bite rate	(Intercept)	1	92	2819.501	< 0.001	69.279 ± 7.693
	Time period	1	92	14.795	< 0.001	See Table 2
	Playback	2	42	1.750	0.186	See Table 2
	Time period × Playback	2	92	3.107	0.049	See Table 2
Step rate	(Intercept)	1	84	235.305	< 0.001	1.104 ± 0.145
	Time period	1	84	4.194	0.044	See Table 2
	Playback	2	41	2.113	0.134	See Table 2
	Time period × Playback	2	84	6.560	0.002	See Table 2
Vigilance while chewing	(Intercept)	1	75	891.392	<.0001	0.983 ± 0.061
	Time period	1	75	1.7833	0.1858	See Table 3
	Playback	2	48	0.8319	0.4414	See Table 3
	Time period × Playback	2	75	5.2121	0.0076	See Table 3
			**Df**	**LRT**	**p-value**	**Coeff ± SE**
Exclusive vigilance	Time period		1	18.505	< 0.001	See Table 3
	Playback		2	69.941	< 0.001	See Table 3
	Time period × Playback		2	13.804	0.001	See Table 3

The proportion of time spent in vigilance was ArcSinSqRoot transformed and step rate and the time spent in vigilance while chewing were log-transformed. See Tables S1, S2, S3 and S4 for details on factors that were controlled for. The pre-playback period and the control playback were used as references for the time period and playback variables, respectively. Vigilance, bite rate, step rate and vigilance while chewing were analyzed using linear mixed-effects models and exclusive vigilance using zero inflated Poisson mixed-effects models (see methods).

### Behavioural Measures during the Pre-Playback Period and Responses to the Control Stimuli

**Figure 1:**
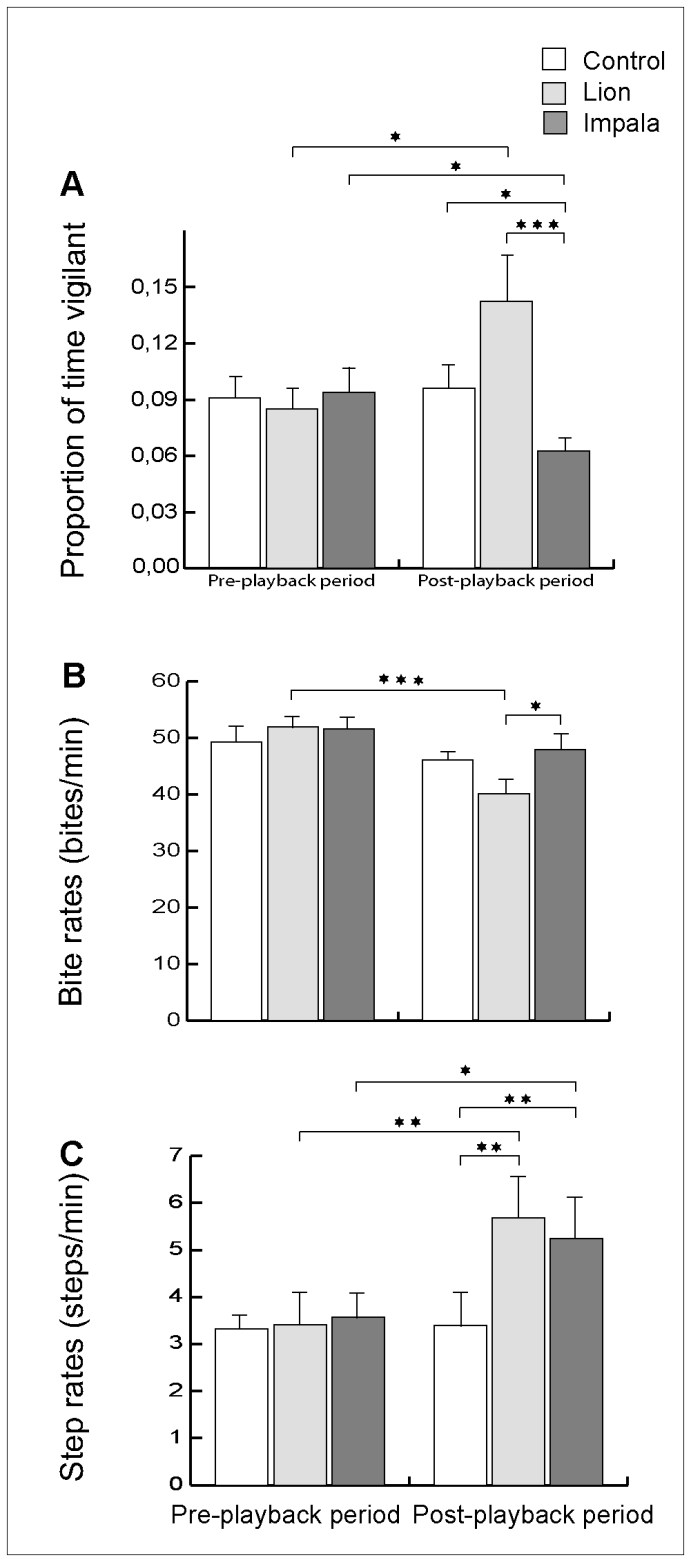
Effects of playbacks on females’ behaviour. Mean (A) proportions of time spent in vigilance (± SE), (B) bite rates (± SE) (numbers of bites per minute during foraging), and (C) step rates (± SE) (numbers of steps per minute) of female impalas exposed to control stimuli, playbacks of lions’ roars and male impalas’ calls during pre- and post-playback periods. *, ** and *** indicate significance at the p < 0.05, p < 0.01 and p < 0.001 levels, respectively.

**Figure 2:**
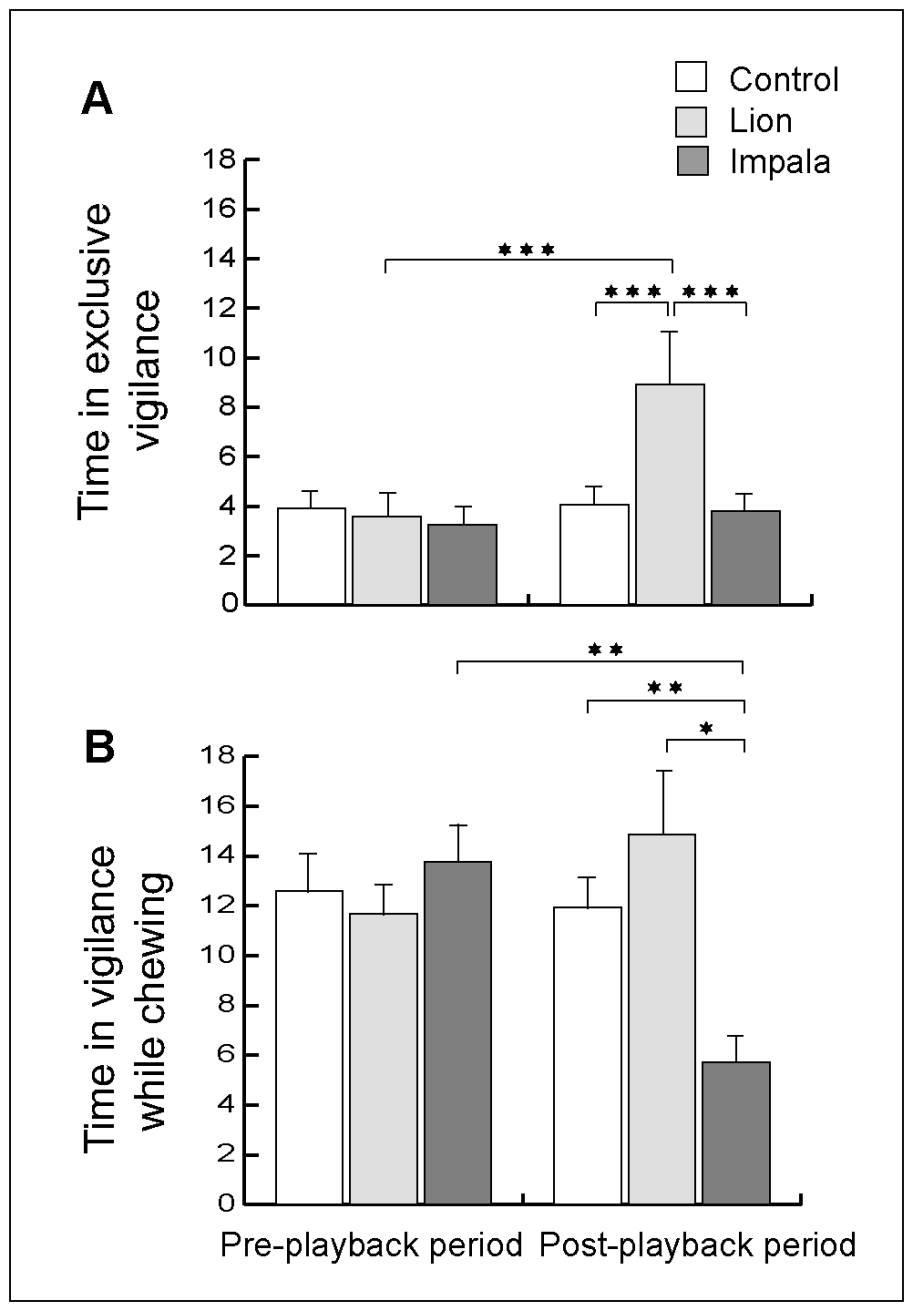
Effects of playbacks on the use of exclusive vigilance and vigilance while chewing. Mean proportions of time (± SE) spent by female impalas in (A) exclusive vigilance and (B) vigilance while chewing during the pre- and post-playback periods after their exposure to playbacks of control stimuli, lions’ roars and male impalas’ calls. *, ** and *** indicate significance at the p < 0.05, p < 0.01 and p < 0.001 levels, respectively.

We found no differences among the three types of playback experiments in the proportions of time devoted to vigilance, the bite rates, the step rates, or times spent in exclusive vigilance and vigilance while chewing of female impalas during the pre-playback period (Table 2, 3). On average, the females spent (± SE) 9 ± 0.01 % of their time during these pre-playback observations in vigilance (including 7 ± 0.01 % in vigilance while chewing and 2 ± 0.01 % in exclusive vigilance), took 50.93 ± 1.82 bites per minute and performed 3.43 ± 0.46 steps per minute (Figure 1, 2). Female impalas did not respond to the control playbacks by changing their proportions of time spent vigilant, their bite or step rates, or their exclusive vigilance and vigilance while chewing, between the pre and post-playback periods (Table 4, 5; Figure 1, 2).

**Table 2:** Statistical results of comparisons between playback treatments for the proportion of time spent in vigilance, bite rate and step rate in the pre- and post-playback periods.

**Periods**	**Contrast pairs**	**Vigilance**			**Bite Rate**			**Step Rate**		
		**Coeff ± SE**	***t***	***P***	**Coeff ± SE**	***t***	***P***	**Coeff ± SE**	***t***	***P***
Pre-playback	Lion *vs.* Control	ns	ns	ns	ns	ns	ns	ns	ns	ns
	Impala *vs.* Control	ns	ns	ns	ns	ns	ns	ns	ns	ns
	Lion *vs.* Impala	ns	ns	ns	ns	ns	ns	ns	ns	ns
Post-playback	Lion *vs.* Control	ns	ns	ns	ns	ns	ns	0.710 ± 0.193	3.672	0.001
	Impala *vs.* Control	-0.083 ± 0.031	-2.653	0.011	ns	ns	ns	0.524 ± 0.194	2.709	0.009
	Lion *vs.* Impala	0.134 ± 0.035	3.855	>0.001	-7.993 ± 2.970	-2.692	0.010	ns	ns	ns

The statistical comparisons included the Holm correction (for multiple comparisons).

**Table 3:** Statistical results of comparisons between playback treatments for the time spent in vigilance while chewing and exclusive vigilance in the pre- and post-playback periods.

**Periods**	**Contrast pairs**	**Vigilance while chewing**			**Exclusive vigilance**		
		**Coeff ± SE**	***t***	***P***	**Coeff ± SE**	***z***	***P***
Pre-playback	Lion *vs.* Control	ns	ns	ns	ns	ns	ns
	Impala *vs.* Control	ns	ns	ns	ns	ns	ns
	Lion *vs.* Impala	ns	ns	ns	ns	ns	ns
Post-playback	Lion *vs.* Control	ns	ns	ns	-0.689 ± 0.099	-6.973	>0.001
	Impala *vs.* Control	0.227 ± 0.096	2.887	0.006	ns	ns	ns
	Lion *vs.* Impala	0.279 ± 0.105	2.654	0.011	0.841 ± 0.110	7.676	>0.001

The statistical comparisons included the Holm correction (for multiple comparisons).

**Table 4:** Comparisons between the pre- and post-playback periods for the proportion of time spent in vigilance, bite rate and step rate for each experimental treatment.

**Playbacks**	**Contrast pairs**	**Vigilance**			**Bite Rate**			**Step Rate**		
		**Coeff ± SE**	***t***	***P***	**Coeff ± SE**	***t***	***P***	**Coeff ± SE**	***t***	***P***
Control	Pre *vs.* Post-playback	ns	ns	ns	ns	ns	ns	ns	ns	ns
Lion	Pre *vs.* Post-playback	-0.070 ± 0.033	-2.141	0.035	11.669 ± 2.767	4.218	>0.001	-0.643 ± 0.194	-3.319	0.001
Impala	Pre *vs.* Post-playback	0.069 ± 0.032	2.173	0.032	ns	ns	ns	-0.405 ± 0.193	-2.102	0.039

The statistical comparisons included the Holm correction (for multiple comparisons).

### Effects of Lion and Impala Playbacks on Vigilance

Female impalas significantly modified their proportions of time spent in vigilance after being exposed to playbacks of both lions’ and impalas’ vocalizations. In the three minutes following the playbacks of lions’ calls, the impalas’ proportion of time vigilant was significantly greater than in the pre-playback period, although post-playback vigilance time after lion and control playbacks did not differ significantly (*P* = 0.120) (Tables 2 and 4, Figure 1A). After being exposed to males’ vocalizations, female impalas significantly decreased their vigilance in comparison with both their responses to the control playbacks during the post-playback period and their level of vigilance during the pre-playback period (Tables 2 and 4, Figure 1A). Group identity did not have a significant effect in any of the analyses (Table S1).  The results of the effects of the other factors that we controlled for are presented in Table S1. 

When analyzing in detail the types of vigilance affected by the stimuli, differentiating between exclusive vigilance and vigilance while chewing, we found that after being exposed to lion playbacks, females significantly increased their exclusive vigilance but did not change their level of vigilance while chewing compared to the pre-playback period and to the control playback during the post-playback period (Tables 3 and 5, Figure 2). Also, when exposed to males’ territorial vocalizations, females significantly decreased their vigilance while chewing but did not change their level of exclusive vigilance compared to either the pre-playback period or the control playback during the post-playback period (Table 3 and 5, Figure 2). The results of the effects of the other factors that we controlled for in the analyses of vigilance while chewing are presented in Table S2.

**Table 5:** Comparisons between the pre- and post-playback periods for the time spent in vigilance while chewing and exclusive vigilance for each experimental treatment.

**Playbacks**	**Contrast pairs**	**Vigilance while chewing**			**Exclusive vigilance**		
		**Coeff ± SE**	***t***	***P***	**Coeff ± SE **	***z***	***P***
Control	Pre *vs.* Post-playback	ns	ns	ns	ns	ns	ns
Lion	Pre *vs.* Post-playback	ns	ns	ns	-0.614 ± 0.114	-5.377	>0.001
Impala	Pre *vs.* Post-playback	-0.343 ± 0.099	-3.457	0.001	ns	ns	ns

The statistical comparisons included the Holm correction (for multiple comparisons).

### Effects of Lion and Impala Playbacks on Bite Rates

Females significantly decreased their bite rates after the lion stimuli compared to the pre-playback period from on average 51 to 40 bites per minutes. During the post-playback period they showed significantly lower bite rates after lions’ roars than after males’ vocalizations, and nearly significantly lower bite rates compared to the control stimuli (*P* = 0.065). In contrast, there was no effect of playbacks of males’ vocalizations on the bite rates of females compared to their bite rates before the playbacks or to those of females exposed to control playbacks (Tables 2 and 4, Figure 1B). The results of the effects of the factors controlled for are presented in Table S3.

### Effects of Lion and Impala Playbacks on Step Rates

After being exposed to both lion and impala playbacks, female impalas significantly increased their step rates compared to females exposed to control stimuli. However, we found no differences between the step rates of females exposed to lions’ roars and females exposed to male impalas’ calls. Females increased their step rates from 3.43 to 5.46 steps per minutes on average (an increase of 59%) in response to these treatments (Tables 2 and 4, Figure 1C). The results of the effects of the factors controlled for are presented in Table S4.

### Testing for Effects of Habituation

While testing for the effects of playbacks, we did not detect any effect of the date of the experiment on either total vigilance (*P*=0.19), exclusive vigilance (*P*=0.22), vigilance while chewing (*P* =0.16), bite rates (*P*=0.15) or step rates (*P*=0.62). Therefore there was no effect of habituation in our experiments.

## Discussion

The results of this study showed that female impalas did not modify any of the measured aspects of their behaviour after hearing the control playbacks, showing that we controlled for potential biases due to the experimental procedures. Also, the females responded differently in terms of both their vigilance and their foraging behaviour to the playbacks of lions’ vocalizations compared with those of male impalas, supporting the hypothesis that they were responding to these particular sounds, rather than just their noise levels. These results show that the female impalas did not react more to the playbacks of lions and impalas than to the control playbacks only because they were louder (loud noise hypothesis, [46]). Because of time constraints and to limit the number of playbacks heard by the impalas (see ethical statement), we did not use a loud noise as a second control; however, given the results we obtained, this form of control was not necessary. In addition, to have comparable playbacks and responses, we only selected and used 15 seconds of each recording. This could be argued to be unrealistic in the case of lions’ roars, which can last between 17 and 90 seconds (reviewed by [45]). However, considering the strong responses of female impalas to our playbacks of lions, the duration of playbacks used during this experiment seems to have been enough to realistically mimic lions’ presence in the vicinity. We strongly believe that our experiments provided solid data for studying the effects of the lions’ and impalas’ vocalizations on the behaviour of wild female impalas. 

Our results revealed that both predators’ and conspecifics’ vocalizations altered the behaviours of social prey species but in different ways. The response of female impalas to playbacks of lions’ roars, in terms of their movements, vigilance and foraging activities, were as predicted. However, their responses to playbacks of males’ vocalizations were strong and not what we had predicted, with animals increasing their movements at the expense of vigilance. These responses highlight the importance of social context to individuals’ behaviours. Finally, we observed that a predator stimulus increased the use of exclusive vigilance while a social stimulus decreased the use of vigilance while chewing. Regardless of the type of stimulus, vigilance while chewing was the main type of vigilance exhibited, which is not surprising considering that herbivores need to optimize their energy intake by limiting the cost of vigilance.

As we expected, our experiment suggests that lions’ roars strongly affect female impalas’ behaviours. After being exposed to the playbacks, females increased their level of vigilance by 40%, on average, with this increase mostly due to an increase in exclusive vigilance. An increase in the vigilance of prey in response to their predators’ vocalizations has already been documented in many species of birds and mammals (reviewed by [22]). Similar results were found by Blanchard & Fritz [20]; although they focused only on the first vigilant bout of impalas in response to playbacks of lions’ roars, they found that alarmed impalas increased their use of exclusive vigilance (which they called “induced vigilance”) compared to non-alarmed animals. Although exclusive vigilance may be more costly for an animal because it stops the ingestion process, it may provide animals with better quality information because chewing is noisy and may reduce the ability of prey to evaluate their predation risk. Thus exclusive vigilance would allow better hearing as well as the stabilization of animals’ visual fields [20]. However, although the relative amount of exclusive vigilance increased, vigilance while chewing remained the major type of vigilance exhibited by females during the post-playback period. This result suggests that impala tend to moderate the foraging cost of vigilance by mainly using a “low-cost” posture of vigilance, even in risky situations. In addition to increasing their vigilance, female impalas increased their step rates by 59% during the post-playback period following lions’ roars. We were not able to control for the positions of individuals (whether they were in the centre or on the edge of their groups) as impala groups were dynamic and their geometry changed frequently, and we did not record the directions of their movements. Nevertheless, we never observed females fleeing in response to the playbacks; they increased their step rates but stayed within their foraging patches. Other studies of ungulates’ movements in predation contexts have suggested that prey may increase their step rates after hearing a predator’s vocalization in order to move to the centre of the group, which is safer ("selfish herd effect" [49]), and bunch together and form denser groups to increase dilution and confusion effects and avoid becoming isolated targets [5,50]. We do not have the data to test these hypotheses; other studies are therefore needed to explore the directions and functions of impalas’ movements in response to predators’ vocalizations.

Finally, female impalas decreased their bite rates after lions’ roars. A decrease in foraging effort under high predation risk has already been observed in many prey species (reviewed by [51]), and can be attributed in our case to the increases in both vigilance and step rates as these activities reduce the time available to take bites. A decrease in bite rates from 51 to 40 bites per minute would seem unlikely to have had nutritional consequences for the impalas. However, our playbacks were short (15 s) and played only once per day. As we know that a lion’s roar can last up to 90 s and that a male may roar 38 to 46 times per night [48], multiple roars may cause meaningful foraging costs to impalas during their nocturnal feeding bouts, which represent between 33 and 42 % of their total daily feeding time [52]. Contrary to our expectations, female impalas decreased their vigilance levels by 38% on average after being exposed to male impalas’ territorial vocalizations. This result was unexpected; we had expected females to increase their vigilance to gather information about the males’ behaviour and the social context. In addition, the few studies that have investigated non-alarm social calls of mammals have reported positive effects on vigilance (e.g. for phee calls of marmosets [27], close calls of meerkats [28] and sexual calls of red deer [31]). However, all of these studies only recorded vigilance activity in the first minute following the playbacks. In our study, the reduction in vigilance was mainly due to a decrease in vigilance while chewing, probably because, as prey, impalas have to maintain a certain level of exclusive vigilance. Although the female impalas spent less time vigilant, they did not increase their bite rates after the males’ vocalizations, but rather increased their movements. We did not record the direction of females’ movements, but Schenkel [30] reported that male impalas’ territorial vocalizations sometimes attracted females but also induced them to bunch together. The decrease in their vigilance may thus have been a result of their increased movements. In addition, it is possible that the impalas’ reaction to the playbacks of males’ vocalizations was much shorter than their reaction in response to the lion stimuli, so that by the time the post-playback period began after the playbacks of males’ vocalizations, the focal males had already stopped being vigilant and were moving to find good feeding positions again, explaining the measured reduction in vigilance. Finally, the males’ vocalizations used in this experiment came from commercial sound archives and therefore did not belong to any males from the studied area. We therefore cannot exclude the possibility that females would have reacted in a different way to the calls of local males. Nevertheless this pattern is interesting and future studies should record the directions of females’ movements, and compare the effects of vocalizations of local and foreign males.

This study investigated the effects of predator and social stimuli on the behavior of social foragers. Our results showed that female impalas reacted to both types of cues in very different ways. While their response to playbacks of predator vocalizations was as expected, the most interesting result concerned their reactions to playbacks of social calls. Males’ territorial vocalizations strongly affected females’ time investment in their main activities. Future studies are needed to gain a better understanding of the ways in which social factors influence vigilance activity in gregarious prey species, differentiating social from antipredator vigilance, and considering the costs of these two types of vigilance (i.e. exclusive vigilance and vigilance while chewing). Although we did not control for the directions in which animals moved in response to both stimuli, these results were highly significant and future studies need to investigate this phenomenon more precisely.

## Supporting Information

Table S1 **Effects of time period and type of playback on the proportion of time spent in vigilance by female impalas (ArcSinSqRoot transformed), controlling for the effects of date, group size (log-transformed), distance to cover and grass height.**
(DOC)

Table S2 **Effects of time period and type of playback on the time spent in vigilance while chewing by female impalas (log-transformed), controlling for the effects of date, group size (log-transformed), distance to cover and grass height.**
(DOC)

Table S3 **Effects of time period and type of playback on the bite rate of female impalas, controlling for the effects of date, group size (log-transformed), distance to cover and grass height.**
(DOC)

Table S4 **Effects of time period and type of playback on the step rate of female impalas (log-transformed), controlling for the effects of individual identity, date, group size (log-transformed), distance to cover and grass height.**
(DOC)
