# Supplementary material for: Short-Term Behavioural Responses of Impalas in Simulated Antipredator and Social Contexts
Source: PLoS One. 2013 Dec 20;8(12):e84970. doi: 10.1371/journal.pone.0084970 (PMC3869902; doi:10.1371/journal.pone.0084970)
Supplement: Table S1 — Effects of time period and type of playback on the proportion of time spent in vigilance by female impalas (ArcSinSqRoot transformed), controlling for the effects of date, group size (log-transformed), distance to cover and grass height. (DOC) [file pone.0084970.s001.doc]

**Table S1.** Effects of time period and type of playback on the proportion of time spent in vigilance by female impalas (ArcSinSqRoot transformed), controlling for the effects of date, group size (log-transformed), distance to cover and grass height.

| **Variables** | **numDF** | **denDF** | **F-value** | **p-value** | **Coeff ± SE** |
| --- | --- | --- | --- | --- | --- |
| (Intercept) | 1 | 100 | 614.309 | < 0.001 | 0.288 ± 0.022 |
| Time period | 1 | 100 | 0.028 | 0.867 | See Table 2 |
| Playback | 2 | 42 | 3.628 | 0.035 | See Table 2 |
| Time period × Playback | 2 | 100 | 4.631 | 0.012 | See Table 2 |
| Date | 1 | 42 | 1.743 | 0.194 |  |
| Log group size | 1 | 42 | 2.914 | 0.095 |  |
| Distance to cover | 4 | 42 | 0.537 | 0.710 |  |
| Grass height | 2 | 42 | 2.651 | 0.082 |  |

Log (Group size) and date were considered as continuous. Time period (*Pre-playback*, post-playback), playback (*Control*, Lions’ roars, Males’ roars), individual identity, distance to cover (0-25, 26-50, 51-100, 101-200, *more than 200m*), grass height (*short*, medium, tall), were categorical (classes used as references are italicized in the legends). Two nested random factors were included, individual within group identity (group identity: *P*=0.229; individual identity: *P*=0.263).
