# Supplementary material for: Short-Term Behavioural Responses of Impalas in Simulated Antipredator and Social Contexts
Source: PLoS One. 2013 Dec 20;8(12):e84970. doi: 10.1371/journal.pone.0084970 (PMC3869902; doi:10.1371/journal.pone.0084970)
Supplement: Table S3 — Effects of time period and type of playback on the bite rate of female impalas, controlling for the effects of date, group size (log-transformed), distance to cover and grass height. (DOC) [file pone.0084970.s003.doc]

**Table S3.** Effects of time period and type of playback on the bite rate of female impalas, controlling for the effects of date, group size (log-transformed), distance to cover and grass height.

| **Variables** | **numDF** | **denDF** | **F-value** | **p-value** | **Coeff ± SE** |
| --- | --- | --- | --- | --- | --- |
| (Intercept) | 1 | 92 | 2819.501 | < 0.001 | 69.279 ± 7.693 |
| Time period | 1 | 92 | 14.795 | < 0.001 | See Table 2 |
| Playback | 2 | 42 | 1.750 | 0.186 | See Table 2 |
| Time period × Playback | 2 | 92 | 3.107 | 0.049 | See Table 2 |
| Date | 1 | 42 | 2.143 | 0.151 |  |
| Log group size | 1 | 42 | 4.427 | 0.041 | -7.782 ± 4.232 |
| Distance to cover | 4 | 42 | 0.748 | 0.565 |  |
| Grass height | 2 | 42 | 5.258 | 0.009 | Medium: -7.509 ± 3.132 (*P* = 0.021) |
|  |  |  |  |  | High: -8.194 ± 3.074 (*P* = 0.011) |

Log (Group size) and date were considered as continuous. Time period (*Pre-playback*, post-playback), playback (*Control*, Lions’ roars, Males’ roars), individual identity, distance to cover (0-25, 26-50, 51-100, 101-200, *more than 200m*), grass height (*short*, medium, tall), were categorical (classes used as references are italicized in the legends). Two nested random factors were included, individual within group identity (group identity: *P*=0.152; individual identity: *P*=0.988).
